# Regional Brain Injury Burden on Term-Equivalent MRI Is Associated with Neurodevelopmental Outcome in Extremely Preterm Infants with Intraventricular Hemorrhage

**DOI:** 10.3390/children13101361

**Published:** 2026-10-08

**Authors:** Julia Buchmayer, Renate Fuiko, Gregor Kasprian, Katrin Klebermaß-Schrehof, Sophie Stummer, Angelika Berger, Katharina Goeral

**Affiliations:** 1 Department of Pediatrics and Adolescent Medicine, Division of Neonatology, Pediatric Intensive Care, and Neuropediatrics, Medical University of Vienna, a full member of ERN EpiCARE, Waehringer Guertel 18-20, 1090 Vienna, Austria; julia.buchmayer@meduniwien.ac.at (J.B.); renate.fuiko@meduniwien.ac.at (R.F.); katrin.klebermass-schrehof@meduniwien.ac.at (K.K.-S.); sophie.stummer@meduniwien.ac.at (S.S.); angelika.berger@meduniwien.ac.at (A.B.); 2 Comprehensive Center for Pediatrics, Medical University of Vienna, Waehringer Guertel 18-20, 1090 Vienna, Austria; 3 Department of Radiology, Division of Neuroradiology and Musculoskeletal Radiology, Medical University of Vienna, 1090 Vienna, Austria; gregor.kasprian@meduniwien.ac.at

**Keywords:** neurodevelopmental outcomes, extremely preterm infant, brain injury, intraventricular hemorrhage, magnetic resonance imaging, neonatal brain injury, neuroimaging, prematurity-related brain injury

## Abstract

**Highlights:**

**What are the main findings?**

- Higher regional cMRI injury burden was associated with poorer cognitive, language, and motor outcomes at two years corrected age.
- Adding the regional cMRI injury score to conventional intraventricular hemorrhage grading significantly improved model fit across all three developmental domains.

**What is the implication of the main finding?**

- In extremely preterm infants with intraventricular hemorrhage, regional evaluation of brain injury may complement conventional intraventricular hemorrhage grading and contribute to a more differentiated assessment of neurodevelopmental risk.

**Abstract:**

Background/Objectives: Extremely preterm infants remain at high risk of adverse neurodevelopmental outcomes, particularly in the presence of intraventricular hemorrhage (IVH) and associated brain injury. Term-equivalent cerebral magnetic resonance imaging (cMRI) provides a detailed assessment of structural brain injury and may provide anatomical information not captured by conventional IVH grading. We aimed to investigate whether the extent and regional distribution of cMRI-detected brain injury are associated with neurodevelopment at two years corrected age. Methods: This retrospective cohort study included extremely preterm infants born before 28 weeks’ gestation with IVH, term-equivalent cMRI, and neurodevelopmental follow-up at two years corrected age. Regional brain injury was assessed on T2-weighted cMRI using a Bassan-derived anatomical approach. Associations with neurodevelopment were examined using multivariable regression models. Results: A total of 137 infants were included in the regional cMRI analysis. The regional injury score ranged from 0 to 10. Each additional affected cerebral region was independently associated with lower cognitive (β −3.62; 95% CI −5.09 to −2.14), language (β −3.13; 95% CI −4.90 to −1.36), and motor scores (β −4.70; 95% CI −6.08 to −3.32), all *p* < 0.001, after adjustment for prespecified clinical and demographic covariates. Associations remained significant after adjustment for conventional IVH classification. Inclusion of the regional cMRI injury score significantly improved model fit for cognitive (*p* = 0.019), language (*p* = 0.006), and motor outcomes (*p* < 0.001). In exploratory region-specific analyses, frontal injury, particularly posterior frontal involvement, showed the largest effect estimates. Conclusions: Higher regional cMRI injury burden was associated with poorer cognitive, language, and motor outcomes in extremely preterm infants with IVH. Exploratory regional assessment suggested that frontal injury was associated with adverse neurodevelopmental outcomes. These findings support the value of considering both the extent and anatomical distribution of brain injury on term-equivalent cMRI when characterizing neurodevelopmental risk.

## 1. Introduction

Extremely preterm infants, born before 28 weeks of gestation, remain at high risk of neurodevelopmental impairment despite major improvements in survival. Cognitive, motor, learning, and behavioral difficulties are common in this population and particularly relevant in infants with neonatal brain injury [1,2,3,4,5].

Intraventricular hemorrhage (IVH) is a major complication in extremely preterm infants and remains associated with considerable morbidity and mortality. It most commonly arises from the germinal matrix next to the lateral ventricles, a highly vascular and fragile region vulnerable to fluctuations in cerebral blood flow [6].

Cerebral ultrasound (CUS) is the standard bedside imaging tool for the detection and grading of IVH in the neonatal intensive care unit [7]. However, cerebral magnetic resonance imaging (cMRI), typically performed around term-equivalent age (TEA), provides a more detailed assessment of brain injury and may detect hemorrhagic lesions with greater sensitivity [8].

Abnormal findings on cMRI at TEA have been consistently associated with adverse neurodevelopmental outcomes [9,10], including in preterm infants with IVH, as demonstrated by Goeral et al. [11]. Conventional IVH grading primarily reflects hemorrhage severity and may not fully capture the extent, laterality, and regional distribution of associated parenchymal brain injury.

Bassan et al. [12] proposed a CUS-based severity score for infants with periventricular hemorrhagic infarction (PVHI) that incorporates lesion extent, bilateral involvement, and midline shift. A higher score is associated with impaired neurodevelopmental outcomes. The use of cMRI may offer the opportunity to quantify injury across predefined cerebral regions and thereby provide a more detailed characterization of overall injury burden.

We therefore investigated whether the extent of regional injury on term-equivalent cMRI was associated with cognitive, language, and motor outcomes in extremely preterm infants with IVH. We also examined whether involvement of specific cerebral regions was related to domain-specific neurodevelopmental outcomes and additionally evaluated the cMRI-reconstructed Bassan score.

## 2. Materials and Methods

### 2.1. Participants

Extremely preterm infants born at 22^6⁄7^ to 27^6⁄7^ weeks of gestation between May 2011 and September 2023 who were admitted to the level IV neonatal intensive care unit at the Medical University of Vienna, had intraventricular hemorrhage identified on serial CUS, underwent cMRI at TEA, and had neurodevelopmental follow-up data available at two years of corrected age were analyzed. IVH was diagnosed and graded on serial CUS examinations according to the classification described by Papile et al. [13]. Cerebral ultrasound examinations were routinely performed on days of life 2, 3 to 4, 7, 14, and 28 and subsequently at regular intervals until discharge. IVH was assessed separately in each hemisphere; in infants with bilateral IVH, the higher grade was used for the conventional IVH classification. PVHI was classified as a separate category rather than as grade IV IVH, according to the classification described by El-Dib et al. [14].

Infants with concomitant cystic periventricular leukomalacia (cPVL) were excluded from the regional analyses to restrict these analyses to IVH-associated injury patterns. Furthermore, infants with major congenital anomalies or known genetic disorders associated with neurodevelopmental impairment were excluded.

Demographic, perinatal, and neonatal clinical data were obtained from the medical records using previously established data collection protocols [8].

### 2.2. cMRI Examinations

All cMRI examinations were performed as part of routine clinical care at the Department of Radiology, Division of Neuroradiology and Musculoskeletal Radiology, using a 1.5-T Philips Ingenia MR system (Philips Healthcare, Best, the Netherlands). The protocol comprised axial T1-weighted spin-echo imaging, sagittal three-dimensional T1-weighted imaging, axial, sagittal, and coronal T2-weighted turbo spin-echo imaging, axial susceptibility-weighted imaging, and axial diffusion-weighted imaging. Detailed acquisition parameters have been published [8]. Since 2017, cMRI at TEA has been routinely offered to all infants born before 28 weeks’ gestation, irrespective of previously detected abnormalities on serial cerebral ultrasound. Before this change in clinical practice, cMRI was mainly performed in infants with grade II IVH complicated by posthemorrhagic ventricular dilation, grade III IVH or PVHI. The relatively high proportion of infants with high-grade IVH and neurodevelopmental impairment in the present cohort reflects this earlier imaging practice. The examinations were carried out according to a local feed-and-wrap protocol, using a vacuum immobilization mattress to minimize motion during image acquisition. A comparable approach has previously been described by Woodward et al. [15].

### 2.3. Regional cMRI

**Figure 1:**
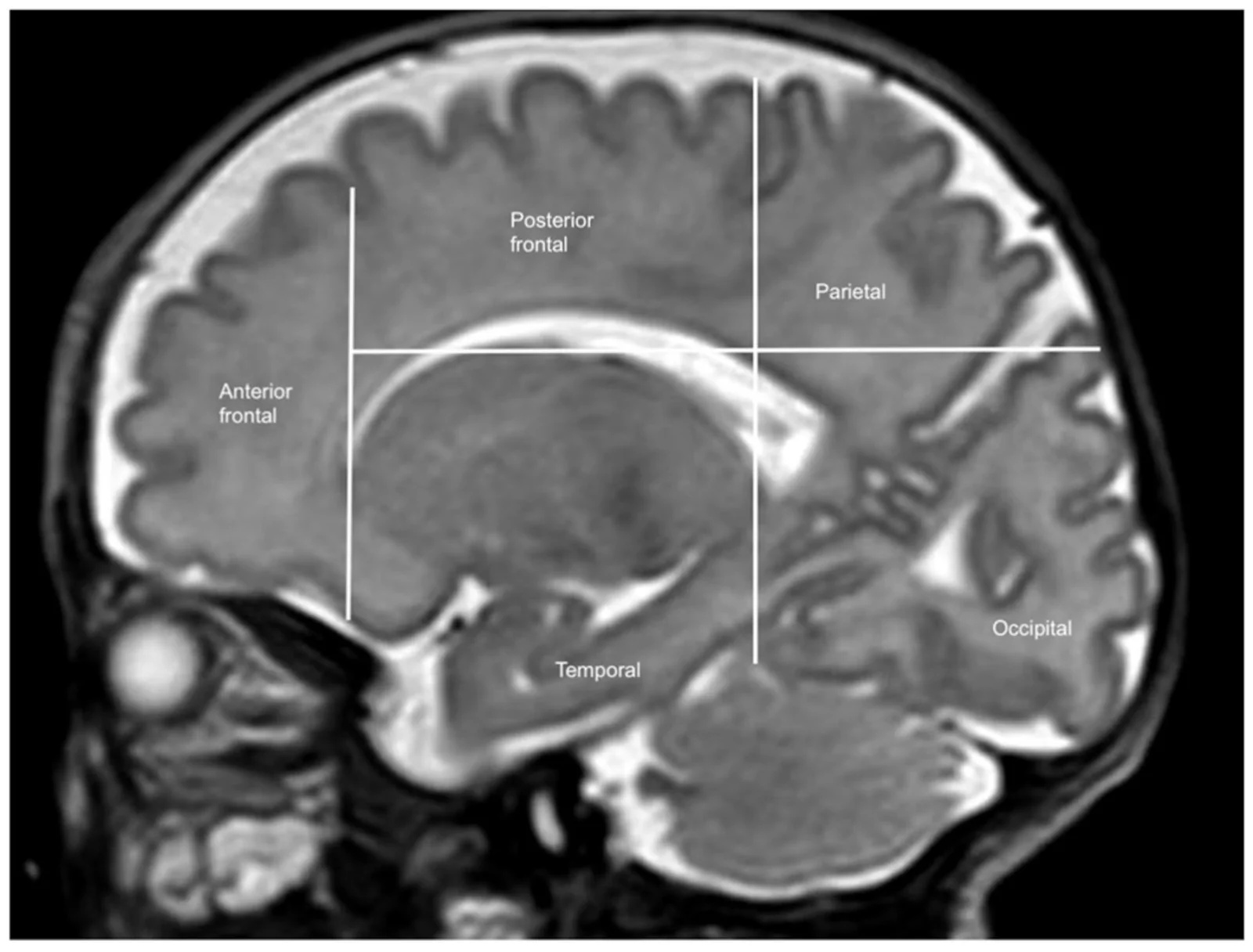
Definition of cerebral regions for regional cMRI injury assessment. Legend: Sagittal T2-weighted cMRI image displaying the five cerebral regions adapted from the anatomical framework described by Bassan et al. [12]—anterior frontal, posterior frontal, parietal, occipital, and temporal—with regional boundaries defined using three anatomical reference lines in relation to the thalamus.

Regional brain injury was assessed on T2-weighted cMRI images in three imaging planes (axial, coronal, and sagittal). Based on the CUS description by Bassan et al. [12], each cerebral hemisphere was divided into five predefined regions: anterior frontal, posterior frontal, parietal, temporal, and occipital. Each region was evaluated separately for the presence of structural injury. The classification system is displayed in Figure 1. The boundaries were defined on the sagittal plane using three anatomical reference lines in relation to the thalamus, adapted from the approach described by Bassan et al. [12]. One horizontal line was drawn along the superior border of the thalamus, and two approximately vertical lines were positioned at its anterior and posterior margins. These reference lines divided each hemisphere into five regions: the anterior frontal region anterior to the anterior reference line, the posterior frontal region superior to the thalamus between the anterior and posterior reference lines and superior to the horizontal reference line, the parietal region posterior to the posterior reference line and superior to the horizontal reference line, the occipital region posterior to the posterior reference line and inferior to the horizontal reference line, and the temporal region inferior to the thalamus/horizontal reference line.

A region was classified as affected when structural parenchymal abnormalities attributable to hemorrhagic injury or local structural consequences were present. This included direct parenchymal hemorrhagic injury as well as visible tissue distortion, thinning, or tissue loss associated or resulting from the hemorrhagic/ventricular lesion. Ventricular dilation without accompanying focal parenchymal abnormality was not considered regional involvement. Other prematurity-related MRI abnormalities that were not anatomically attributable to the IVH-related lesion, including diffuse white matter signal abnormalities, generalized cerebral volume reduction, delayed maturation, or isolated cerebellar abnormalities, were not included in the regional score.

Regional involvement was recorded independently for the right and left hemispheres. The number of affected regions was summed for each hemisphere, resulting in a score ranging from 0 to 5 per side. For exploratory region-specific analyses, laterality was not considered and a cerebral region was considered involved if injury was present in either hemisphere. The total regional cMRI injury score ranged from 0 to 10, with higher scores indicating a greater extent of cerebral injury.

In addition, the original CUS-based Bassan score [12] was reconstructed using cMRI. Points were assigned for extensive involvement (≥two regions), for bilateral injury, and for the presence of a midline shift. The three components were combined to calculate a Bassan score [12] ranging from 0 to 3. This cMRI-reconstructed score has not been validated as a cMRI-based scoring system and was used only to examine whether the components of the original Bassan score were associated with neurodevelopmental outcome.

All cMRI assessments were performed blinded to neurodevelopmental outcomes, prior cerebral ultrasound and clinical data. Regional cMRI assessments were performed by two senior neonatologists with extensive experience in neonatal neuroimaging, and a senior neonatal neuroradiologist. Disagreements were resolved by consensus. Intra- and interrater reliability of the total regional cMRI injury score was assessed in a randomly selected subset of 15% of all examinations according to Brennan and Silman [16].

### 2.4. Neurodevelopmental Outcome

Infants born extremely preterm were routinely invited to a standardized neurodevelopmental follow-up assessment at two years corrected age. Cognitive, language, and motor development were assessed using the Bayley Scales of Infant and Toddler Development, Third Edition (Bayley-III) [17], with scores derived from the German normative data. Developmental assessments were performed by certified clinical psychologists experienced in the evaluation of former preterm infants.

### 2.5. Statistical Analyses

Statistical analyses were performed using R version 4.5.2 (R Foundation for Statistical Computing, Vienna, Austria). Continuous variables are presented as median (interquartile range) and categorical variables as number (percentage). Missing values were handled using a complete-case approach. The number of observations included in each regression model is reported together with the respective model results. Associations between regional cMRI injury scores and neurodevelopmental outcomes were examined using linear regression. Unadjusted models included the regional cMRI injury score as the sole predictor. Adjusted models additionally included gestational age at birth, birth weight percentile, sex, bronchopulmonary dysplasia, retinopathy of prematurity requiring intervention, patent ductus arteriosus requiring surgical or interventional closure, and necrotizing enterocolitis with surgery. These covariates were prespecified based on established associations with neurodevelopmental outcome in extremely preterm infants. Potential nonlinearity of the association between regional cMRI injury score and neurodevelopmental outcomes was explored in additional sensitivity analyses by including a quadratic term for the regional cMRI injury score in the adjusted regression models. The shape of the association was also assessed visually using LOESS curves, which were used for visualization only.

To account for changes in MRI practice over time, additional analyses were stratified by imaging era (before 2017 vs. 2017 onward), and a sensitivity analysis was performed restricting the cohort to infants born from 2017 onward.

Effect modification by PVHI status was assessed by including an interaction term between PVHI status and the regional cMRI injury score in the adjusted models.

To assess whether regional cMRI injury burden was associated with neurodevelopmental outcomes independently of conventional IVH classification, nested linear regression models were constructed separately for cognitive, language, and motor outcomes. Model 1 included the same prespecified demographic and clinical covariates described above. Model 2 additionally included conventional IVH classification, entered as a categorical variable, and Model 3 further included the regional cMRI injury score. Nested models were compared using ANOVA F tests. Changes in adjusted R^2^ were reported to quantify improvement in model fit after inclusion of the regional cMRI injury score. These analyses were intended to assess independent association and incremental model fit rather than to develop or validate an individual prediction model.

To assess the association between injury location and outcome, region-specific analyses were adjusted for involvement of the remaining cerebral regions and gestational age at birth. Region-specific analyses were considered exploratory. Therefore, no formal adjustment for multiple comparisons was applied.

The cMRI-reconstructed Bassan score was analyzed using linear regression adjusted for the same prespecified demographic and clinical covariates as the primary adjusted model.

All tests were two-sided, and a *p*-value < 0.05 was considered statistically significant.

## 3. Results

### 3.1. Study Cohort

In the study period a total of 1132 infants <28 weeks’ gestation were born, and the study cohort consisted of 146 infants with term-equivalent cMRI and available neurodevelopmental follow-up data (displayed as a flow diagram in the Supplemental Figure S1).

Median gestational age at birth was 25^2/7^ weeks (24^1/7^–26^3/7^), and median birth weight was 770 g (610–890). The cMRI examinations were performed at a median postmenstrual age of 38^1/7^ weeks (37^0/7^–39^4⁄7^). The IVH grades were I in 21 (14.4%), II in 42 (28.8%), and III in 45 (30.8%), and PVHI was present in 38 (26.0%) infants. Further neonatal and clinical characteristics are presented in Table 1.

Bayley-III cognitive scores were available in 130 infants with a median of 70 (IQR 55–85), language scores were available in 130 infants with a median of 69 (IQR 45–84), and motor scores were available in 137 infants with a median of 73 (IQR 49–89).

Data were complete for all covariates included in the primary adjusted models except for retinopathy of prematurity requiring intervention, for which data were unavailable in 2 infants (1.5%). Consequently, the primary adjusted analyses included 128 infants for cognitive and language outcomes and 135 infants for motor outcome.

### 3.2. Regional cMRI Injury Characteristics

**Figure 2:**
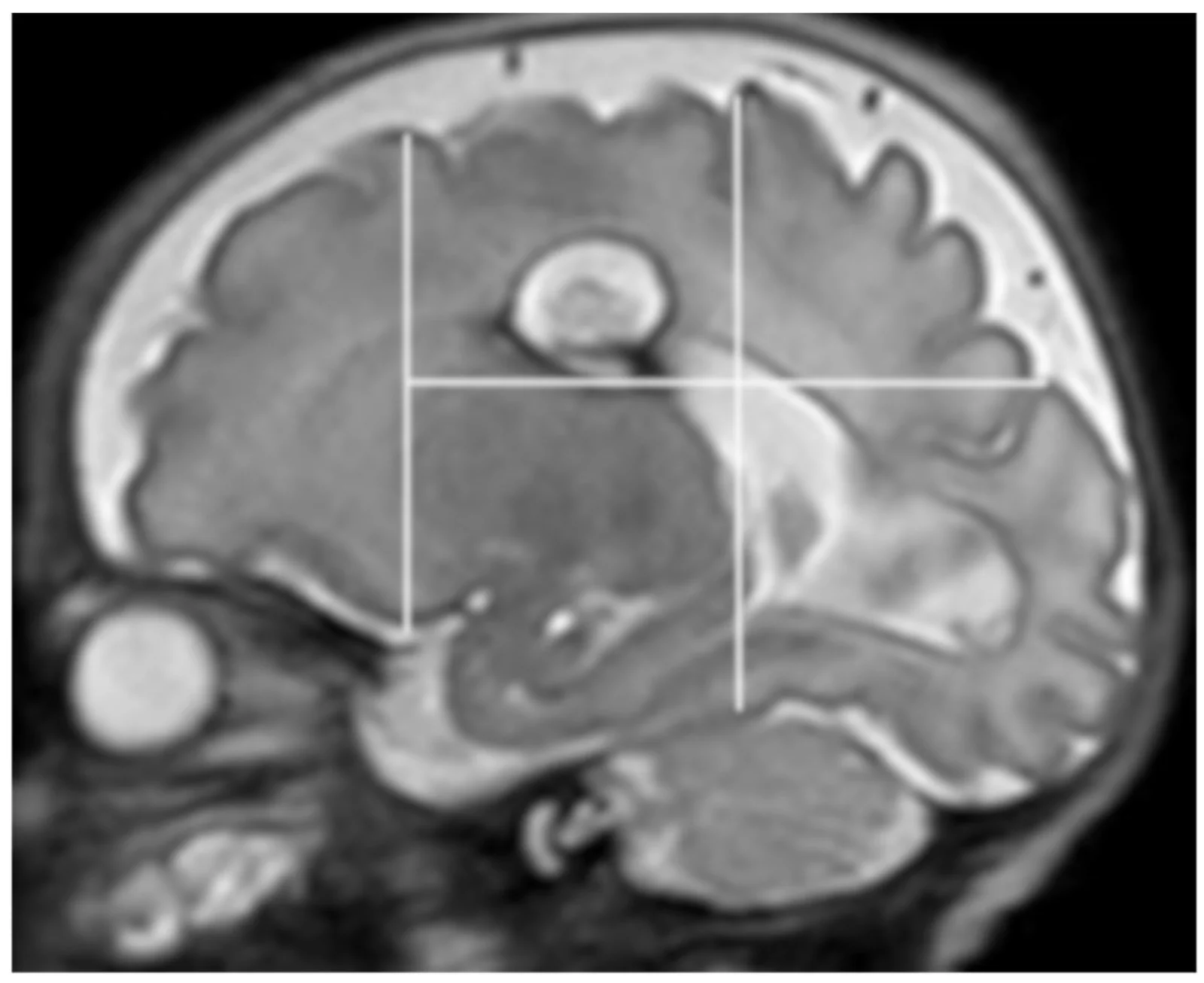
Example of regional cMRI brain injury assessment. Legend: Sagittal T2-weighted cMRI image with isolated posterior frontal involvement in one hemisphere resulting in a regional cMRI injury score of 1.

**Figure 3:**
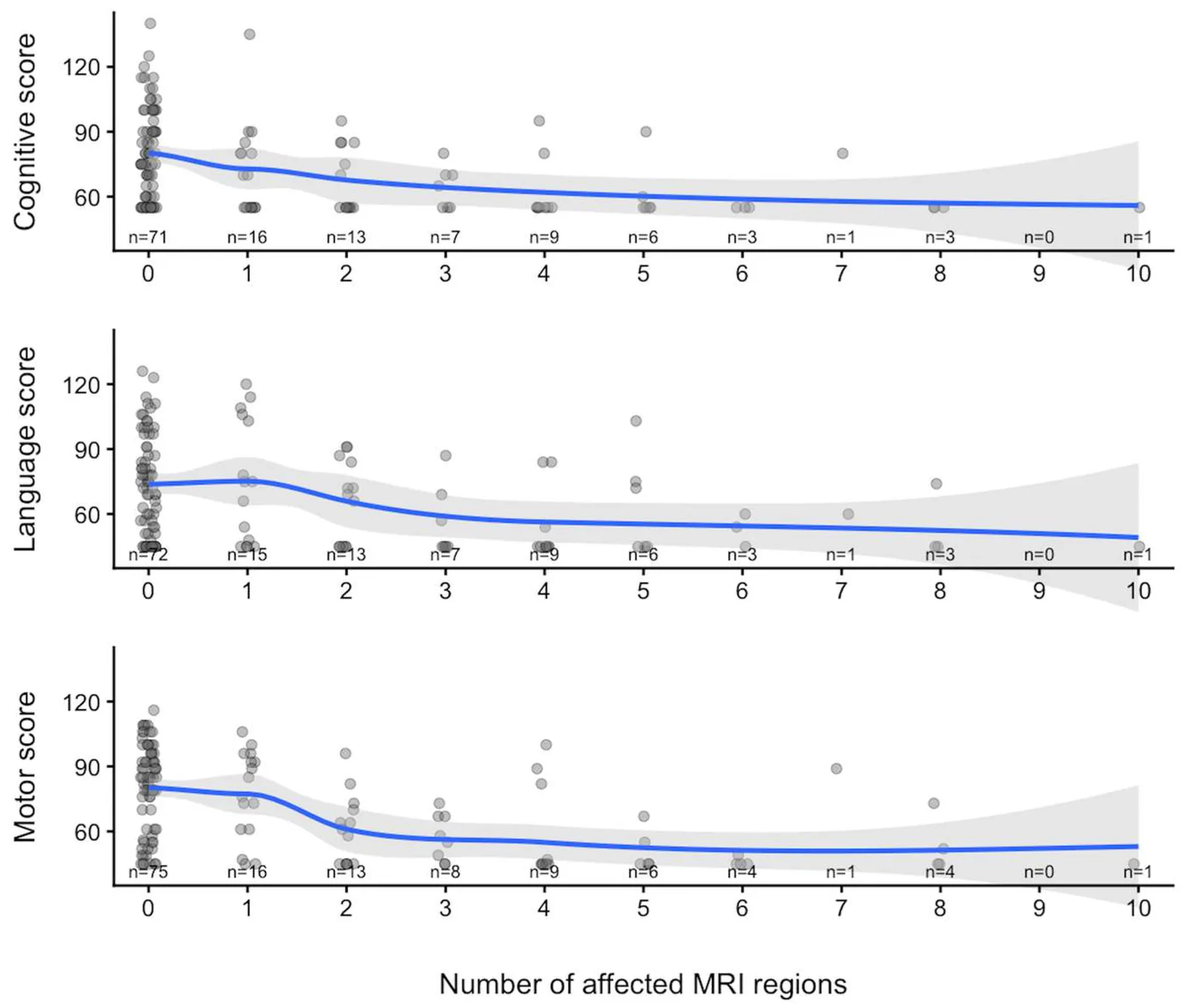
Association between cumulative regional cMRI injury burden and neurodevelopmental outcome. Legend: Individual points represent the composite scores of the included infants according to the number of affected cerebral regions on TEA cMRI, the blue line represents locally estimated scatterplot smoothing (LOESS), and the shaded area indicates the 95% confidence interval. LOESS curves were used for visualization only and were not used for inferential analyses.

**Figure 4:**
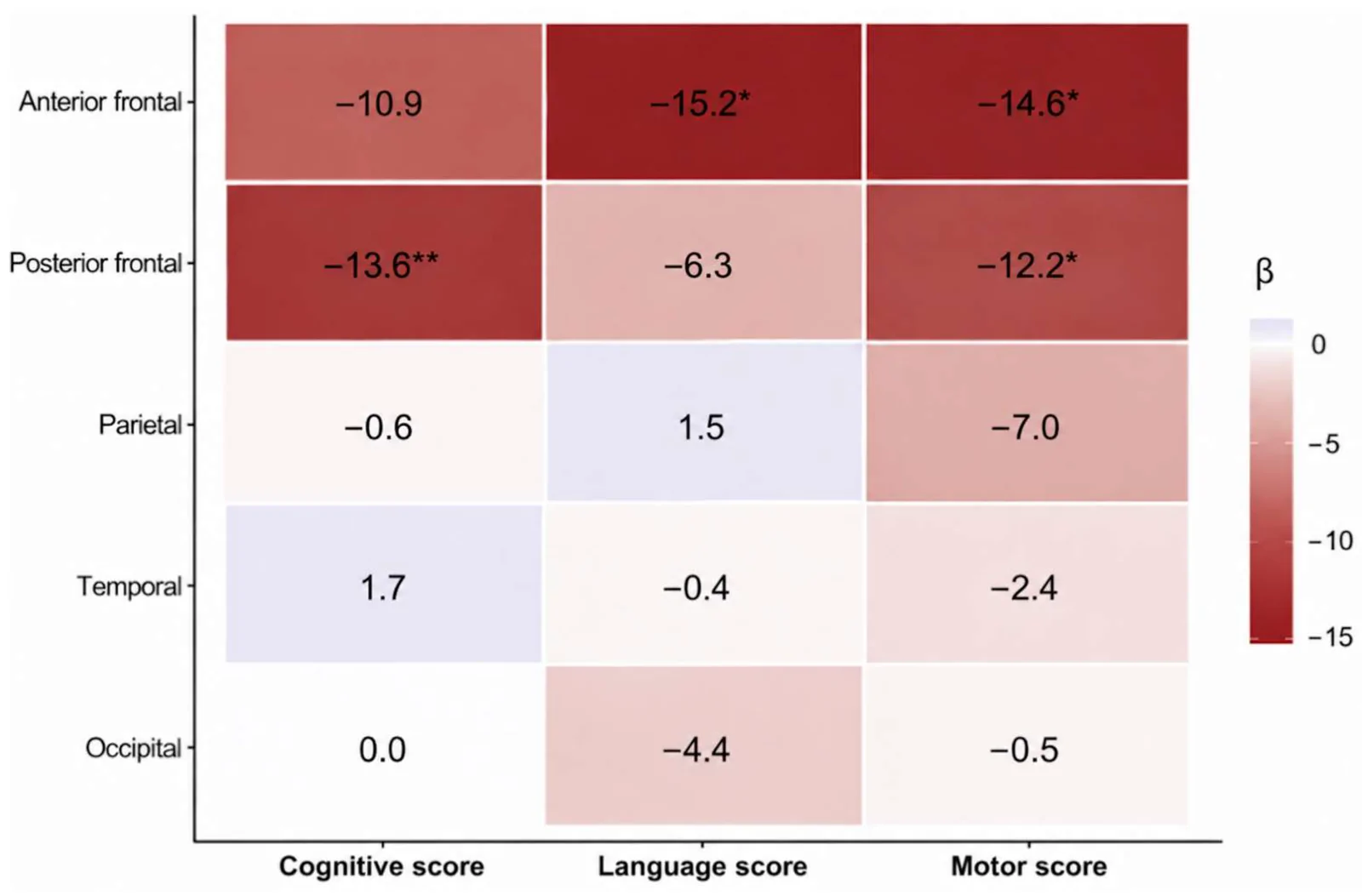
Regional cMRI injuries and neurodevelopmental outcomes. Legend: Heatmap showing adjusted β coefficients for the associations between involvement of five predefined cerebral regions and Bayley-III cognitive, language, and motor composite scores; adjustment for other involved brain regions and gestational age at birth; * *p* < 0.05, ** *p* < 0.01.

After exclusion of infants with cPVL, 137 infants were included in the final cMRI analyses. The regional cMRI injury score ranged from 0 to 10, with a median score of 0 (IQR 0–2). No regional injury was identified in 75 infants (54.7%), while 62 infants (45.3%) had involvement of at least one region. Scores of 1 (displayed in Figure 2), 2, 3, 4, and 5 were observed in 16 (11.7%), 13 (9.5%), 8 (5.8%), 9 (6.6%), and 6 (4.4%) infants. More extensive injury involving six or more regions was present in 10 infants (7.3%).

Intra- and interrater reliability of the total regional cMRI injury score was assessed in a randomly selected subset of 15% of all examinations and showed a very good agreement according to Brennan and Silman [16], with an interrater ICC of 0.899 (95% CI 0.830 to 0.952) and intrarater ICC of 0.986 (95% CI 0.965 to 0.994).

### 3.3. Association of Cumulative Regional cMRI Injury Score and Neurodevelopmental Outcomes

A higher cumulative regional cMRI injury score was independently associated with lower cognitive, language, and motor scores at two years corrected age. In adjusted analyses, each additional affected cerebral region was associated with a lower cognitive score (β −3.62; 95% CI −5.09 to −2.14, *p* < 0.001), lower language score (β −3.13; 95% CI −4.90 to −1.36, *p* < 0.001), lower motor score (β −4.70; 95% CI −6.08 to −3.32; *p* < 0.001), as displayed in Table 2.

Additional sensitivity analyses suggested some deviation from strict linearity for cognitive and motor outcomes. The quadratic term was significant for cognitive (β 0.62; 95% CI 0.06 to 1.18; *p* = 0.031) and motor outcomes (β 0.95; 95% CI 0.44 to 1.46; *p* < 0.001), but not for language outcome (β 0.30; 95% CI −0.39 to 0.99; *p* = 0.390). The primary linear regression coefficients were therefore interpreted as average associations across the observed score range.

The association between regional cMRI injury burden and neurodevelopmental outcome was also observed when analyses were stratified by imaging era. In a sensitivity analysis restricted to infants born from 2017 onward (n = 91), higher regional injury scores remained significantly associated with lower cognitive, language, and motor scores (all *p* < 0.001).

Infants with PVHI had a higher regional injury burden, with a median regional cMRI injury score of 3 (IQR 1–5) in infants with PVHI compared with 0 (IQR 0–1) in those without PVHI. However, there was no evidence that PVHI status modified the association between regional injury score and cognitive (β −0.80; 95% CI −4.50 to 2.90; *p* = 0.669), language (β −2.04; 95% CI −6.48 to 2.40; *p* = 0.365), or motor outcome (β −2.27; 95% CI −5.72 to 1.17; *p* = 0.194).

As illustrated in Figure 3, the cumulative regional cMRI injury score showed an inverse association with neurodevelopmental performance. Cognitive, language, and motor scores decreased with increasing numbers of affected cMRI regions. The decline was most pronounced for motor outcome, with lower scores observed as regional injury burden increased. At higher regional injury scores, confidence bands widened because of the smaller number of infants with extensive injuries.

After inclusion of conventional IVH classification and the prespecified demographic and clinical covariates, the regional cMRI injury score remained independently associated with cognitive, language, and motor outcomes. The addition of the regional cMRI injury score to models including demographic and clinical covariates and conventional IVH classification improved model fit for cognitive (adjusted R^2^ 0.284 to 0.311; F(1.116) = 5.62; *p* = 0.019; n = 128), language (adjusted R^2^ 0.078 to 0.128; F(1.116) = 7.78; *p* = 0.006; n = 128), and motor outcomes (adjusted R^2^ 0.391 to 0.443; F(1.123) = 12.47; *p* < 0.001; n = 135). Each additional affected cerebral region remained independently associated with lower cognitive (β −2.12; 95% CI −3.89 to −0.35), language (β −3.14; 95% CI −5.37 to −0.91), and motor scores (β −2.82; 95% CI −4.40 to −1.24).

### 3.4. Association of Regional cMRI Injury Location and Neurodevelopmental Outcomes

The associations between involvement of the five cerebral regions and cognitive, language, and motor outcomes are displayed in Figure 4. After adjustment for injury burden in the remaining cerebral regions and gestational age at birth, posterior frontal involvement was associated with a significantly lower cognitive score (β −13.60; 95% CI −21.70 to −5.51; *p* = 0.001). Anterior frontal involvement showed a negative association with cognitive score (β −10.90; 95% CI −22.20 to 0.42; *p* = 0.059) that did not reach statistical significance. Furthermore, the anterior frontal region was associated with a lower language score (β −15.20; 95% CI −28.70 to −1.77; *p* = 0.027). For motor outcome, both anterior frontal (β −14.60; 95% CI −25.00 to −4.18; *p* = 0.006) and posterior frontal involvement (β −12.20; 95% CI −20.10 to −4.28; *p* = 0.003) were significantly associated with lower scores. No associations for parietal, temporal, or occipital regions reached nominal statistical significance. Given the multiple correlated comparisons and absence of multiplicity correction, these findings should be interpreted cautiously. Complete effect estimates and 95% confidence intervals for all regions are provided in Supplementary Table S1.

### 3.5. cMRI-Reconstructed Bassan Score

The cMRI-reconstructed Bassan score [12] ranged from 0 to 3 and was available in 135 of 137 infants. In two infants, midline shift could not be reliably assessed on cMRI, so they were excluded from this analysis. A score of 0 was present in 89 infants (65.9%), a score of 1 in 16 (11.9%), a score of 2 in 18 (13.3%), and a score of 3 in 12 (8.9%). In the adjusted analyses, each one-point increase in Bassan score was associated with significantly lower cognitive (β −8.03; 95% CI −11.2 to −4.84; *p* < 0.001), language (β −7.34; 95% CI −11.1 to −3.59; *p* < 0.001), and motor (β −10.7; 95% CI −13.8 to −7.62; *p* < 0.001) outcome scores.

## 4. Discussion

In this study, we examined how the extent and anatomical distribution of periventricular cerebral injury on term-equivalent cMRI were associated with neurodevelopmental outcome in extremely preterm infants with IVH. Increasing regional injury burden was consistently associated with lower cognitive, language, and motor scores at two years corrected age, with the strongest association for motor outcome. In addition, frontal involvement, particularly posterior frontal injury, showed the largest exploratory region-specific effects. The cMRI-reconstructed Bassan score [12] was also associated with cognitive, language, and motor outcomes. This simple regional assessment of the extent and distribution of structural brain injury may provide a more detailed anatomical characterization of injury patterns associated with later neurodevelopment. Conventional IVH grading remains an important tool in neonatal practice and provides relevant prognostic information. However, it primarily describes the severity of the hemorrhage and ventricular involvement and may not fully capture the extent and anatomical distribution of associated parenchymal injury. Abnormal findings on TEA cMRI have repeatedly been associated with adverse neurodevelopmental outcome in preterm infants, including specifically those with IVH [10,11].

This distinction may be particularly relevant in infants with PVHI, in whom the anatomical extent of tissue injury can vary considerably despite the same conventional IVH classification. In additional analyses, infants with PVHI had a higher regional cMRI injury burden than those without PVHI, but there was no evidence of effect modification by PVHI status. In the present cohort, the number of affected cerebral regions was strongly associated with neurodevelopmental outcome. After adjustment for demographic and clinical covariates, each additional affected region was associated with a reduction in cognitive, language, and motor scores. The association was strongest for motor development, which may partly reflect the frequent involvement of periventricular tissue in hemorrhagic and post-hemorrhagic injuries and the close proximity of developing corticospinal and other motor pathways [18,19]. At the same time, the associations with cognitive and language scores demonstrate that the consequences of IVH-related injury are not restricted to motor function.

The relationship was not strictly linear across the entire score range, particularly for cognitive and motor outcomes, suggesting that the effect of additional affected regions may vary with increasing injury burden. Accordingly, the coefficients from the linear models should be interpreted as average effects across the observed range rather than as a constant effect for each additional region.

Importantly, the regional cMRI injury score remained independently associated with cognitive, language, and motor outcomes after adjustment for demographic and clinical covariates and conventional IVH classification. Moreover, adding the regional cMRI injury score improved model fit across all three developmental domains, suggesting that regional injury burden captures outcome-relevant information not fully reflected by conventional IVH classification alone. These analyses assess model fit rather than clinical predictive performance.

An important part of this study was the assessment of injury location. After accounting for injury burden in the remaining cerebral regions and gestational age at birth, frontal involvement showed the largest exploratory region-specific effect estimates. Posterior frontal injury was associated with substantially lower cognitive and motor scores, while anterior frontal involvement was associated with language and motor outcomes. The posterior frontal region includes developing motor and premotor areas and lies close to major descending white matter pathways. Furthermore, frontal regions contribute to higher-order cognitive, language, motor-planning, and behavioral functions [18,19,20,21]. In preterm brains, the immature periventricular white matter and developing frontal-subcortical networks may be particularly vulnerable to hemorrhagic injury and its effects, which could contribute to the observed associations. In addition, the anatomical distribution of tissue injury following IVH may preferentially affect fibers and networks traversing these regions during a critical period of brain development [19,22,23]. Conversely, the weaker associations observed for parietal, temporal, and occipital involvement may reflect differences in lesion frequency, extent, or functional consequences rather than true regional differences in vulnerability. However, these analyses involved multiple correlated comparisons without multiplicity correction and should therefore be regarded as hypothesis-generating rather than confirmatory.

Bassan et al. [12] developed a CUS severity score for PVHI incorporating lesion extent, bilateral involvement, and midline shift. When this cMRI-reconstructed Bassan score was applied in our cohort, each additional score point was associated with a significant reduction in cognitive, language and motor scores.

Our regional approach was based on the same anatomical principle and provided a more detailed description of cerebral involvement using cMRI. The purpose of this approach was to separate overall injury burden from the location of injury. Regional information may provide further context, particularly in infants with complex injury patterns. Such information may provide additional anatomical context for neurodevelopmental risk assessment. However, clinical application of the regional scoring approach requires further reliability testing and validation in independent cohorts.

Future studies should evaluate whether regional cMRI assessment can contribute to multimodal risk-stratification models for extremely preterm infants with IVH, alongside conventional IVH classification and established clinical predictors. A more detailed characterization of injury extent and anatomical distribution may ultimately help identify infants at increased neurodevelopmental risk who could benefit from closer surveillance or earlier supportive interventions. However, the present findings do not establish clinical predictive utility. Although intra- and interrater reliability was very good in the present cohort, reproducibility across different centers and raters requires further evaluation. Prospective multicenter studies with larger cohorts, external validation, and longer-term neurodevelopmental follow-up are needed to determine whether incorporation of the regional score improves prediction and clinical utility sufficiently to support its use in prognostic algorithms or counseling.

### Strengths and Limitations

A strength of this study is the relatively large cohort of extremely preterm infants with IVH who underwent cMRI at TEA and had standardized neurodevelopmental follow-up at two years corrected age. An advantage of the regional assessment is that it is simple and quick to apply and does not require additional post-processing.

Several limitations should be considered. This was a retrospective single-center study, and cMRI practice changed during the study period. Before routine cMRI was introduced in 2017, imaging was preferentially performed in infants with more severe IVH or post-hemorrhagic ventricular dilatation, resulting in enrichment of the cohort for more severe brain injury and adverse neurodevelopmental outcome and limiting generalizability to contemporary unselected populations of extremely preterm infants with IVH.

Infants with concomitant cPVL were excluded to focus the regional analyses on IVH-associated injury patterns. The findings may not apply to infants with mixed hemorrhagic and cystic white matter injury. Several potential sources of residual confounding were not systematically available for the entire cohort, including detailed family history of neurodevelopmental disorders, prenatal exposure to medications or substances of abuse, congenital coagulation disorders, and maternal or peripartum infectious exposures.

Region-specific analyses were exploratory and involved multiple correlated comparisons without formal adjustment for multiplicity. These findings should therefore be interpreted cautiously.

The Bassan score [12] was originally developed for CUS assessment of PVHI and was retrospectively reconstructed from cMRI in the present study. It should not be considered equivalent to or a validation of the original ultrasound-based score. In particular, features such as midline shift may evolve between the acute neonatal period and TEA and may therefore not be captured equivalently by the two imaging approaches.

Neurodevelopmental outcome was assessed at two years corrected age using the Bayley-III test which captures early developmental performance, but does not fully predict later cognitive, executive, behavioral, or academic functioning.

The present analyses assessed associations rather than the predictive performance of a prognostic model, and the regional scoring approach requires validation in an independent cohort.

## 5. Conclusions

The extent of cerebral injury on term-equivalent cMRI was strongly associated with neurodevelopmental outcome in extremely preterm infants with IVH. A greater number of affected regions was associated with lower cognitive, language, and particularly motor scores at two years corrected age. Frontal injury, especially posterior frontal involvement, showed the largest exploratory region-specific effect estimates. Regional assessment of term-equivalent cMRI provides a simple way of describing the anatomical burden of IVH-related cerebral injury and may contribute to a more differentiated assessment of neurodevelopmental risk.

## Figures and Tables

**Table 1:** Neonatal and clinical characteristics.

Characteristics	Study Cohort (n = 146)	Regional Analysis Cohort (Without cPVL) (n = 137)
GA at birth (weeks)	25^2/7^ (24^1/7^–26^3/7^)	25^2/7^ (24^1/7^–26^3/7^)
Cesarean delivery	109 (74.7)	100 (73.0)
Multiple births	39 (26.7)	36 (26.3)
Antenatal steroids	125 (85.6)	117 (85.4)
Male sex	91 (62.3)	86 (62.8)
Birthweight (g)	770 (610–890)	770 (610–900)
Birthweight percentile	51 (28–69)	49 (27–67)
1-min Apgar score	7 (5–8)	7 (5–8)
5-min Apgar score	8 (7–9)	8 (7–9)
10-min Apgar score	9 (8–9)	9 (8–9)
pH_umb_	7.34 (7.29–7.38)	7.34 (7.29–7.38)
pH_neon_	7.18 (7.09–7.24)	7.18 (7.09–7.24)
Lactate_neon_ (mmol/L)	4.6 (3.5–7.4)	4.6 (3.6–7.4)
Culture-proven sepsis	44 (30.1)	41 (29.9)
IVH severity		
I	21 (14.4)	21 (15.3)
II	42 (28.8)	37 (27.0)
III	45 (30.8)	43 (31.4)
PVHI	38 (26.0)	36 (26.3)
cPVL	9 (6.2)	0 (0.0)
Patent ductus arteriosus with surgical or interventional closure	18 (12.3)	15 (10.9)
Necrotizing enterocolitis with surgery	9 (6.2)	8 (5.8)
Retinopathy of prematurity with intervention	34 (23.3)	34 (24.8)
Bronchopulmonary dysplasia (oxygen requirement ≥36 weeks of gestation)	67 (45.9)	62 (45.3)

Legend: Data are displayed as median (interquartile range) or n (%) as appropriate. Abbreviations: GA, gestational age; IVH, intraventricular hemorrhage; Lactate_neon_, first neonatal lactate; pH_neon_, first neonatal pH after birth; pH_umb_, umbilical artery pH; PVHI, periventricular hemorrhagic infarction.

**Table 2:** Association of the regional cMRI injury score with neurodevelopmental outcomes at corrected two years of age.

Outcome	Model	β (95% CI)	*p*	Adjusted R^2^
Cognitive score	Unadjusted	−3.45 (−4.98 to −1.92)	<0.001	0.128
	Adjusted	−3.62 (−5.09 to −2.14)	<0.001	0.251
Language score	Unadjusted	−3.13 (−4.90 to −1.37)	<0.001	0.081
	Adjusted	−3.13 (−4.90 to −1.36)	<0.001	0.137
Motor score	Unadjusted	−4.45 (−5.90 to −2.99)	<0.001	0.207
	Adjusted	−4.70 (−6.08 to −3.32)	<0.001	0.343

Legend: β is the mean difference in the respective Bayley-III score per additional affected cerebral region on cMRI. The adjusted model included gestational age at birth, birth-weight percentile, sex, bronchopulmonary dysplasia, retinopathy of prematurity requiring intervention, patent ductus arteriosus requiring surgical or interventional closure, and necrotizing enterocolitis with surgery. Abbreviations: CI, confidence interval.

## Data Availability

The data that support the findings of this study are available from the corresponding author upon reasonable request. The data are not publicly available due to privacy and ethical restrictions related to patient data.

## Supplementary Materials

The following supporting information can be downloaded at https://www.mdpi.com/article/10.3390/children13101361/s1, Figure S1: Participant flow diagram; Table S1: Association of cerebral region-specific injury with neurodevelopmental outcomes at two years corrected age.

## Abbreviations

The following abbreviations are used in this manuscript:

Bayley-III	Bayley Scales of Infant and Toddler Development, 3rd edition
cMRI	Cerebral magnetic resonance imaging
cPVL	Cystic periventricular leukomalacia
CUS	Cerebral ultrasound
IVH	Intraventricular hemorrhage
PVHI	Periventricular hemorrhagic infarction
TEA	Term-equivalent age
