# Supplementary material for: Regional Brain Injury Burden on Term-Equivalent MRI Is Associated with Neurodevelopmental Outcome in Extremely Preterm Infants with Intraventricular Hemorrhage
Source: Children (Basel). 2026 Oct 8;13(10):1361. doi: 10.3390/children13101361 (PMC13703412; doi:10.3390/children13101361)
Supplement: Supplementary file 1 [file children-13-01361-s001.zip › children-4564740-supplementary.pdf]

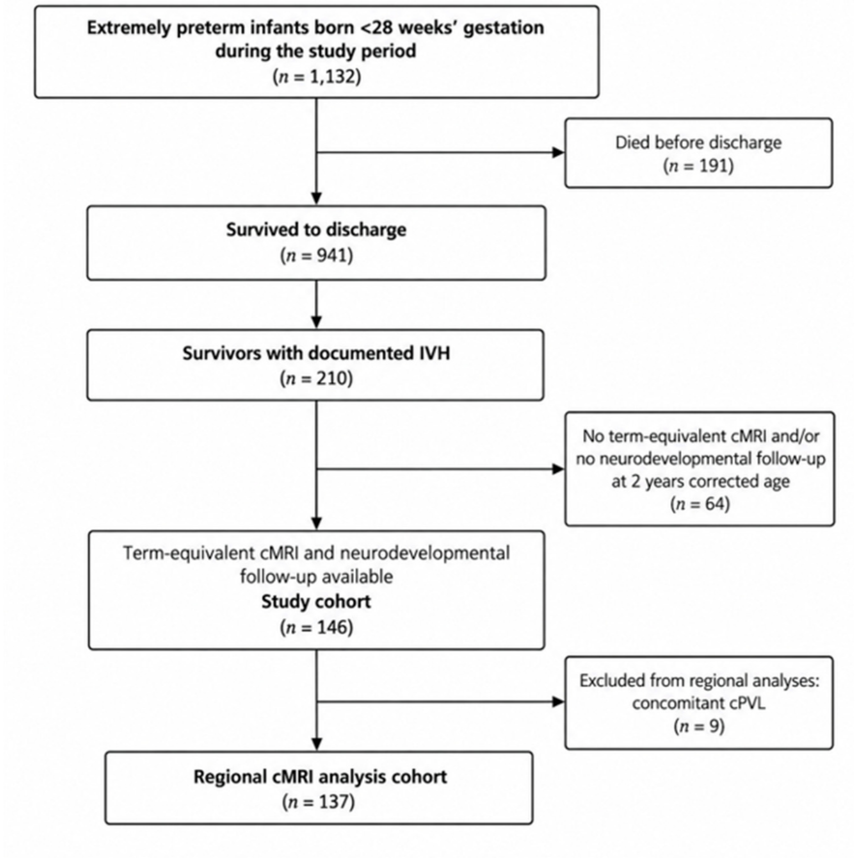

Supplemental Figure S1. Participant flow diagram

Supplementary Table S1. Association of cerebral region-specific injury with neurodevelopmental outcomes at two years corrected age.

Cerebral region	n	Cognitive β (95% CI)	p	Language β (95% CI)	p	Motor β (95% CI)	p
Anterior frontal	19	−10.9 (−22.2 to 0.4)	0.059	−15.2 (−28.7 to −1.8)	0.027	−14.6 (−25.0 to −4.2)	0.006
Posterior frontal	61	−13.6 (−21.7 to −5.5)	0.001	−6.3 (−16.3 to 3.7)	0.216	−12.2 (−20.1 to −4.3)	0.003
Parietal	34	−0.6 (−10.6 to 9.3)	0.903	1.5 (−10.4 to 13.4)	0.802	−7.0 (−16.6 to 2.6)	0.152
Temporal	15	1.7 (−11.1 to 14.4)	0.797	−0.4 (−15.8 to 14.9)	0.956	−2.4 (−14.2 to 9.5)	0.694
Occipital	24	0.0 (−10.5 to 10.6)	0.995	−4.4 (−17.1 to 8.3)	0.494	−0.5 (−10.4 to 9.3)	0.912

Legend: n represents the number of infants with involvement of the specified cerebral region and β represents the adjusted mean difference in the respective Bayley-III composite score associated with involvement of the specified cerebral region. Models were adjusted for injury burden in the remaining cerebral regions and gestational age at birth. Cognitive and language analyses included n=128 infants; motor analyses included n=135 infants. Region-specific analyses were exploratory, and no formal adjustment for multiple comparisons was applied. Abbreviations: CI, confidence interval.
